# Current status and future prospects of non-toxicity carbon-dot-based miniaturized lasers

**DOI:** 10.1093/nsr/nwaf426

**Published:** 2025-09-29

**Authors:** Yongqiang Zhang, Xiao Zeng, Xinyi Wen, Shurong Ding, Yuzhuo Yang, Yongsheng Hu, Siyu Lu

**Affiliations:** College of Chemistry, Pingyuan Laboratory, Zhengzhou University, Zhengzhou 450001, China; College of Chemistry, Pingyuan Laboratory, Zhengzhou University, Zhengzhou 450001, China; College of Chemistry, Pingyuan Laboratory, Zhengzhou University, Zhengzhou 450001, China; College of Chemistry, Pingyuan Laboratory, Zhengzhou University, Zhengzhou 450001, China; College of Chemistry, Pingyuan Laboratory, Zhengzhou University, Zhengzhou 450001, China; School of Physics and Microelectronics, Zhengzhou University, Zhengzhou 450001, China; College of Chemistry, Pingyuan Laboratory, Zhengzhou University, Zhengzhou 450001, China

**Keywords:** miniaturized lasers, solution-processable gain media, carbon dots, non-toxicity, biocompatibility

## Abstract

With rapid advances in on-chip photonic integration and flexible optoelectronic devices, miniaturized laser devices are increasingly desired for various fields including optical communication, biosensing and biomedicine. However, a major barrier to their industrial deployment is the development of solution-processable gain media that are affordable, non-toxic and highly stable. Conventional solution-processable gain media often exhibit high toxicity and limited optical stability, considerably restricting their scalability and practical use. Carbon dots (CDs), as emerging solution-processable gain media, offer a compelling solution for developing environmentally friendly miniaturized lasers owing to their simple synthesis, affordability, non-toxicity, high biocompatibility, superior stability and excellent optical properties. This review summarizes progress in CD-based lasers with diverse resonator configurations, elucidates their gain mechanisms in the context of CD luminescence theories, and proposes strategies to optimize laser performance. Furthermore, it discusses current application prospects alongside critical challenges that must be addressed to advance CD-based miniaturized lasers. Overall, this review highlights the promising nature of CD-based miniaturized lasers and is anticipated to drive further in-depth research in this field.

## Teaser

This review summarizes the research progress, gain mechanism and material optimization strategies of CD-based lasers, and looks forward to their future applications and challenges.

## INTRODUCTION

Since the invention of the first laser in 1960, lasers have become indispensable across a wide range of critical fields, including communication, medicine, manufacturing and scientific research, substantially driving societal advancement [1–5]. As quantum devices that transform input energy into highly coherent optical output, lasers are fundamentally composed of three primary components: the pump source, the gain medium and the resonator. Among these, the pump source delivers the necessary energy to the device, while the resonator provides optical feedback and confines the photon waveform produced via stimulated emission. Meanwhile, the gain medium represents the core element of the laser. It converts external pump energy into highly coherent laser light through stimulated emission [6–8]. As the principal medium responsible for optical amplification, the physical and optical characteristics of the gain medium directly affect key laser performance parameters, including output power, beam quality, energy conversion efficiency and operational stability over time [9]. Consequently, detailed investigations into gain media are not only central to laser physics but also critical to advancing the broad deployment of laser technologies across sectors such as communication, manufacturing, medicine and research [10–12].

With the growing demand for devices that offer easy processability, miniaturization, integration and flexibility, laser technology faces increasing technical pressure to overcome the inherent limitations of conventional architectures [13–15]. Traditional lasers struggle with size reduction owing to physical constraints such as resonator length and challenges in heat dissipation. This limitation has become a critical technical bottleneck, hindering progress in on-chip photonic integration and the development of flexible optoelectronic devices [16–18]. In response, the search for novel gain media tailored for miniaturized lasers has emerged as a prominent area of research. Among these media, solution-processable gain media represent a promising breakthrough for advancing the miniaturization and flexibility of laser systems. Unlike conventional gain materials, solution-processable media allow for the direct preparation of homogeneous, stable solutions by dissolving precursors in solvents. This approach eliminates the need for high-temperature, high-pressure processing and complex lithographic techniques, thereby simplifying fabrication while considerably lowering production costs [19]. Furthermore, the resulting solutions can be readily transformed into films through affordable techniques such as spin coating, inkjet printing or roll-to-roll printing. These methods impart excellent flexibility, enabling the materials to conform to curved or irregular surfaces. Such features open new avenues for applying miniaturized lasers in next-generation fields, including flexible optoelectronics and implantable medical devices [20].

Currently, most solution-processable gain media include organic dyes, colloidal quantum dots and perovskite materials. However, each of these materials faces critical limitations requiring immediate attention. Organic dyes, for instance, require complex synthesis procedures that frequently produce toxic by-products [21]. Additionally, during optical amplification, these dyes undergo substantial thermal degradation and are susceptible to photobleaching, necessitating large-scale cooling systems for stabilization [22,23]. This severely constrains their potential for miniaturization. While colloidal quantum dots and perovskite materials have garnered considerable attention for their high luminescence efficiency and tunable emission wavelengths, their widespread incorporation of heavy metals such as lead and cadmium raises safety concerns for laser operators, and complicates waste management [24–26]. Perovskite materials, in particular, exhibit poor chemical stability in the presence of water and air, which has emerged as a critical bottleneck limiting their practical applications [27]. Therefore, the development of a new class of solution-processable gain media featuring simple synthesis, environmental compatibility and robust stability is essential for advancing miniaturized lasers from the research stage toward practical, industrial deployment.

Carbon dots (CDs) are zero-dimensional nanomaterials with dimensions below 10 nm, consisting of a carbon-based core surrounded by a surface shell enriched with various functional groups [28,29]. As an emerging class of carbon-based nanomaterials, CDs can be synthesized using diverse methods, including hydrothermal treatment, microwave irradiation and laser ablation. Their synthesis is relatively simple and cost-effective, and the precursors are readily available, often derived from natural biomass such as leaves and fruit peels, which generally results in low toxicity and excellent biocompatibility [30,31]. The ‘green’ characteristics of CDs have enabled them to demonstrate certain advantages in the fields of biological imaging, biological sensing and biological therapy. Specifically, in biological imaging and biosensing, CDs can replace traditional quantum dots as probes for early disease detection and cell imaging diagnosis [32]. In biological therapy, the biocompatibility of CDs enables them to have excellent compatibility in drug delivery [33], photodynamic therapy [34] and photothermal therapy [35].

Furthermore, CDs exhibit excellent optical properties, including high photostability, tunable emission wavelengths and a high photoluminescence (PL) quantum yield (QY) [36–38]. Thus far, laser emission has been successfully achieved from CD-based lasers across the visible to near-infrared spectrum, and these materials have also been applied in optical imaging and information storage. These observations highlight the high chemical stability, low toxicity and favorable solution processability of CDs, reinforcing their potential for a wide range of applications in the advancement of miniaturized laser technologies [39]. Table S1 presents some representative laser performance data [40–42]. Compared with organic dyes and colloidal quantum dots, CDs have demonstrated a considerable level in laser performance, while also featuring low cost, excellent photothermal stability and good biocompatibility. Therefore, CDs, as a solution-processable gain medium, demonstrate broad application potential.

This review provides a comprehensive overview of the current research landscape, gain mechanisms, optimization strategies and application prospects of solution-processable miniaturized CD lasers. It begins by summarizing recent advances in CD laser development, with a focus on the resonance mechanisms of different microcavity structures and their implementation in CD laser research. It then explores the gain mechanisms of CDs in detail and outlines strategies to enhance their gain performance from three key perspectives: fluorescence properties; structural design; and resonant cavity configuration. Finally, by integrating the current application landscape with future development trends, this review examines the development trajectory and associated challenges of CD-based solution-processable miniaturized lasers and offers informed insights to guide future research.

## RESEARCH STATUS OF CD LASERS

With the growing strategic relevance of solution-processable miniaturized lasers in optoelectronics, conventional materials are increasingly unable to meet practical application demands owing to their intrinsic toxicity and limited stability. In this context, CDs have rapidly gained attention as a promising new class of functional optical materials, owing to their straightforward synthesis, excellent biocompatibility and markedly lower environmental toxicity [43]. To date, laser emission from CDs has been realized under various resonant modes, including random lasers lacking conventional cavity structures, as well as stimulated emission supported by different optical resonant cavity configurations such as whispering gallery mode (WGM) resonators, Fabry–Perot (F–P) cavities, and distributed Bragg reflector (DBR) cavities. These advances have not only deepened the understanding of the fundamental gain mechanisms of CD lasers but have also led to substantial improvements in the performance of CD-based miniaturized lasers, thereby accelerating their transition toward practical applications.

### Random lasers

**Figure 1.**
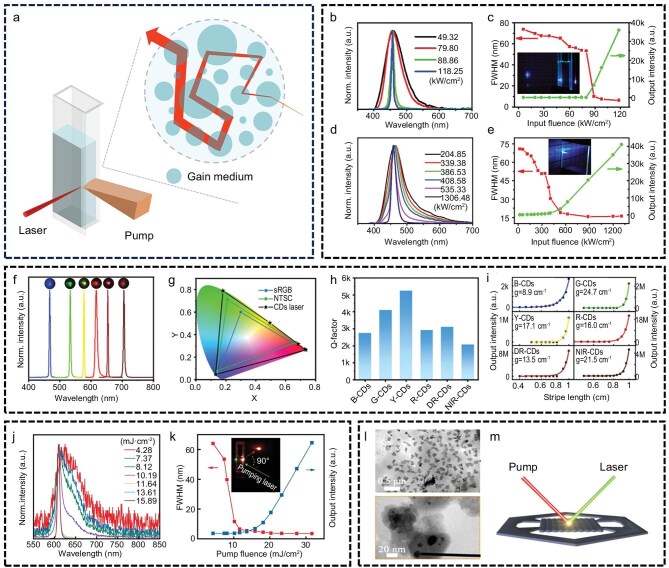
Random laser without external scatterers. (a) CD random laser concept. (b‒e) Random lasing from CDs with excitation wavelength independence. Reprinted with permission from ref [46]. Copyright 2016 Elsevier. (f‒i) Full-color CDs with random lasing from bright blue to near-infrared. B-CD, blue-emitting CD; G-CD, green-emitting CD; Y-CD, yellow-emitting CD; R-CD, red-emitting CD; DR-CD, deep-red-emitting CD; NIR-CD, near-infrared CD. Reprinted with permission from ref [41]. Copyright 2023 Wiley. (j and k) Random lasing from solution-processable vortex lasers. Reprinted with permission from ref [47]. Copyright 2024, Youke Publishing Co., Ltd. (l and m) Random laser emission of acid-triggered aggregated sheet-like CDs. Reprinted with permission from ref [48]. Copyright 2023 Wiley.

Random lasers are a class of laser emission systems that operate without traditional optical resonators. Their defining characteristic is a broad-spectrum or multi-peaked emission profile, resulting from the absence of a mode-selection mechanism. When the pump source injects energy into the gain medium, the medium becomes excited and emits photons. These photons are amplified through repeated interactions with the gain medium and multiple scattering events within the system, ultimately producing optical amplification [44,45]. In this process, the high optical gain of the medium compensates for the lack of a resonator, diminishing its necessity. Notably, the particulate nature of CDs imparts them with inherent light-scattering capability. When combined with the strong optical gain properties of CDs, this ability renders them suitable for constructing random laser systems that function without external scatterers or resonant structures (Fig. 1a). Liu *et al.* synthesized two types of CDs with distinct optical behaviors from the same precursor and found that CDs exhibiting excitation-independent fluorescence were more conducive to random laser emission than their excitation-dependent counterparts. They further demonstrated random laser emission from solid-state films composed of excitation-independent CDs, offering a valuable reference for the development of practical, low-threshold CD laser systems (Fig. 1b‒e) [46]. Lu *et al.* successfully synthesized full-color CDs with fluorescence emission spanning from bright blue to near-infrared and achieved random laser emission via optical pumping in a mirrorless system [41]. This system exhibited an ultra-wide color gamut covering 140% of the NTSC color space and was applied in laser imaging and holographic display technologies. Their research broadened the application scope of CD-based lasers and advanced their progress toward practical deployment (Fig. 1f‒i). In a separate effort, the team demonstrated flexible control over topological charge and mode structure in vortex beams produced by conventional CD random lasers, thereby enriching the class of solution-processable vortex lasers and contributing to the realization of wavelength-tunable vortex emission (Fig. 1j and k) [47]. Additionally, Lu’s group synthesized CDs capable of reversible self-assembly regulated through acid–base interactions and observed that the sheet-like structures formed upon aggregation enabled random laser emission [48]. This pH-responsive structural assembly offers a novel pathway for investigating the gain mechanism in CD lasers (Fig. 1l and m). Collectively, these studies demonstrate that CDs possess excellent performance potential in random laser systems without external scatterers, underscoring their suitability as an ideal random laser medium and providing valuable insights for the development of miniaturized, cavity-free laser devices.

### Random lasers with scattering particles

The multiple scattering behavior of CDs can be further enhanced by incorporating additional scattering agents. Commonly used scatterers include SiO_2_, TiO_2_ and TiN nanoparticles and gold–silver nanowires (Fig. S1a). Wang *et al.* reported that CDs synthesized using bio-derived resorcinol as the sole precursor produced deep-blue random laser emission under the strong scattering of SiO_2_ particles when optically pumped [49]. Their study revealed that the rigid structure formed during synthesis resulted in narrowband emission, offering potential advantages for laser performance (Fig. S1b‒S1d). Messina and colleagues synthesized green-light-emitting CDs and employed them as the gain medium. Assisted by TiO_2_ nanoparticles, they successfully realized random laser emission [50]. Notably, they observed that varying the CD concentration induced a marked transition in the emission behavior—from incoherent to coherent modes (Fig. S1e‒S1j). Yang *et al.* introduced gold and silver porous nanowires as efficient scatterers into blue, green and red CD solutions, enabling the construction of color-tunable random laser systems. These systems not only exhibited ultralow emission thresholds across the visible spectrum but also demonstrated excellent long-term output stability [51]. The development of such low-threshold, high-stability CD lasers provides a strong foundation for future practical applications (Fig. S1k‒S1m). In another study, Liu *et al.* doped TiN nanoparticles into CD-based composite nanowire materials. Benefiting from the plasmonic resonance and scattering effects of these nanoparticles, the luminescence efficiency of CDs was notably enhanced, resulting in a low-threshold, narrowband and stable random laser output [52]. This study presents an effective strategy for developing simple-to-fabricate, affordable and environmentally friendly laser systems (Fig. S1n‒S1p). Although the incorporation of external scatterers can enhance the overall gain efficiency by extending the photon scattering paths within the gain medium, it also introduces drawbacks such as optical losses and system instability due to scattering. Miniaturization offers greater design flexibility and improved environmental tolerance for these scatterers, thereby providing a viable approach to increasing the net gain of the laser system.

### WGM lasers

**Figure 2.**
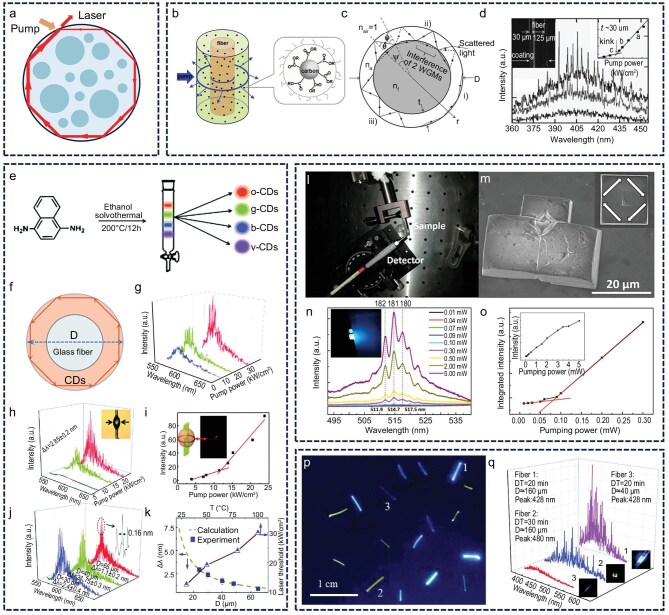
WGM laser. (a) WGM laser concept. (b‒d) Deep-blue WGM lasing using CDs as the gain medium coating on the surface of an optical fiber. Reprinted with permission from ref [54]. Copyright 2012 Wiley. (e‒k) Orange emissive CD-based low-threshold WGM microcavity laser with excellent thermal stability. Reprinted with permission from ref [49]. Copyright 2019 Royal Society of Chemistry. (l‒o) WGM lasing from CD–NaCl hybrid crystals’ microcubic structure. Reprinted with permission from ref [56]. Copyright 2017 American Chemical Society. (p and q) Random lasing CD fibers for multi-level anti-counterfeiting. Reprinted with permission from ref [57] Copyright 2021 Royal Society of Chemistry.

WGM cavities amplify light primarily through total internal reflection at the microcavity surface. When the optical gain provided by the gain medium exceeds the intrinsic losses, photons circulate within the cavity to form a closed feedback loop, enabling laser emission from the interior of the microcavity (Fig. 2a) [53]. Yu *et al.* employed a solution impregnation technique to fabricate a CD/polyethylene glycol composite coating on the surface of an optical fiber, achieving deep-blue WGM lasing using CDs as the gain medium. The coating thickness varied along the axial direction of the fiber, and a clear correlation was observed between the laser mode spacing (Δ*λ*) and the coating thickness [54]. This study represented the first demonstration of CDs as gain media in laser systems, sparking further interest in CD-based lasers (Fig. 2b‒d). In 2019, Yu’s group synthesized high-brightness, narrowband orange-emitting CDs and developed a WGM microcavity laser. By adjusting the microcavity size, they were able to tune the mode spacing, ultimately achieving a low-threshold WGM laser with excellent thermal stability [55]. This study introduced a promising strategy for designing high-intensity, narrowband CDs for use as gain media, thereby advancing the development of high-performance laser technologies (Fig. 2e‒k). Zhao *et al.* fabricated a hybrid crystal by embedding CDs into an NaCl matrix and leveraged its self-assembled microcubic structure to form a WGM optical resonator, thereby achieving directional laser emission [56]. However, this system exhibited a relatively high lasing threshold due to the accumulation of triplet excitons from phosphorescence, which suppressed the stimulated emission process (Fig. 2l‒o). In a related study, Zhang’s group prepared a CD/polystyrene (PS) composite via one-step thermal decomposition and fabricated an optical fiber by stretching the material. The laser emission threshold and wavelength were found to depend on the decomposition time and fiber diameter, introducing a new strategy for multi-level anti-counterfeiting applications. However, the larger fiber diameter compared with that of typical WGM microcavities led to random rather than WGM laser emission (Fig. 2p and q) [57]. These findings underscore that oversized WGM cavities increase optical losses and weaken resonator performance. Therefore, miniaturization of laser structures is critical for improving device efficiency and emission stability.

### F‒P lasers

**Figure 3.**
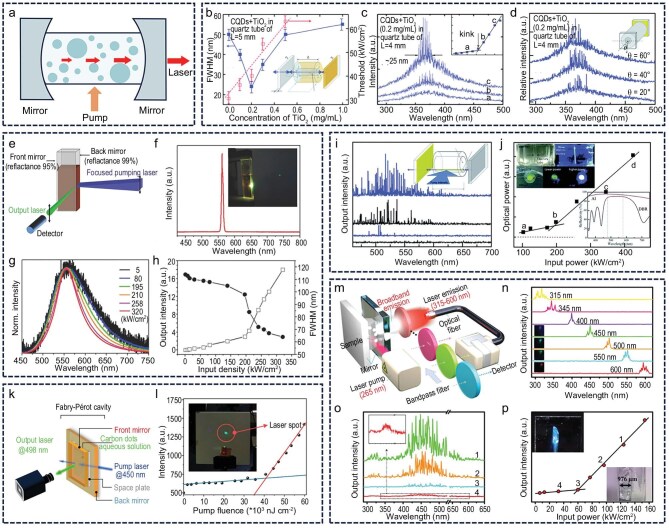
F–P laser. (a) F–P laser concept. (b‒d) Laser emission by integrating functionalized CDs into an F–P cavity. Reprinted with permission from ref [59]. Copyright 2013 Royal Society of Chemistry. (e‒h) High fluorescence QY green-emitting CDs achieved stable laser output in the F–P cavity. Reprinted with permission from ref [60]. Copyright 2013 Wiley. (i and j) Low-threshold solid-state F–P laser of surface-functionalized CDs. Reprinted with permission from ref [61]. Copyright 2014 Royal Society of Chemistry. (k and l) CDs with near 100% QY in aqueous solution for lasing. Reprinted with permission from ref [62]. Copyright 2024 Wiley. (m‒p) Multi-mode laser output based on CD–crystal hybrid material in F–P resonator. Reprinted with permission from ref [42]. Copyright 2022 Wiley.

The F–P cavity, a classical optical resonator structure, forms a feedback loop using a pair of parallel high-reflectivity mirrors. When the gain medium is excited, photons reflect multiple times between the mirrors, undergoing amplification, and coherent laser emission is eventually emitted through a partially reflective mirror (Fig. 3a) [58]. However, conventional F–P cavities are prone to alignment-induced losses, which critically limit their efficiency. Scaling down the cavity to the micro- or nanoscale can effectively reduce these alignment-related losses, making miniaturization a key direction in F–P cavity development. Yu *et al.* realized laser emission by integrating functionalized CDs into an F–P cavity [59]. Owing to their high specific surface area and small volume, the CDs substantially enhanced laser performance, establishing them as high-quality luminescent materials with potential for biological applications (Fig. 3b‒d). Shen *et al.* precisely tuned the heteroatom doping levels in CDs and successfully synthesized blue- and green-emitting variants [60]. Further investigation revealed that the green-emitting CDs, owing to their high fluorescence QY and minimal spectral overlap between absorption and emission in ethanol–water solution, served as efficient gain media within the F–P cavity and enabled stable laser output (Fig. 3e‒h). Yu’s research revealed a synergistic enhancement in luminescence performance achieved by combining surface-functionalized CDs with organic silane groups. The resulting solid-state F–P laser exhibited a lasing threshold two orders of magnitude lower than previous systems and achieved 60 nm tunable single-mode output under the Littrow configuration, introducing a novel material platform for white laser development (Fig. 3i and j) [61]. Qu *et al.* designed CDs with a specialized core–shell structure via solid-state reactions. These materials exhibited a fluorescence QY approaching 100% in aqueous solution. A spatial barrier mechanism, induced by the hydrophobicity of the conjugated units, effectively suppressed water-induced fluorescence quenching, making this the first CD-based material to realize aqueous-phase laser emission within an F–P cavity [62]. This approach, which resists quenching through conjugated structural design, offers a promising pathway for the biological application of CD lasers (Fig. 3k and l). Using a one-pot *in situ* composite method, Xie *et al.* developed a CD/crystal hybrid material system that exhibited strong broadband optical gain within an F–P resonator and supported multi-mode laser output based on CD composites (Fig. 3m‒p) [42].

### DBR lasers

The DBR cavity achieves high reflectivity at specific wavelengths through the periodic stacking of materials with differing refractive indices. Similar to the F–P cavity, the DBR cavity enables multiple light resonances via the reflective interfaces of its multi-layer structure. Owing to its micro–nano-scale design, it facilitates stronger localization of light field resonances, thereby substantially enhancing laser emission (Fig. S2a) [63]. Hang *et al.* embedded CDs *in situ* into a silica network matrix, effectively suppressing intermolecular interactions and non-radiative transitions caused by solid-state aggregation. This was achieved through the 3D framework of the silica network, which markedly improved the fluorescence QY. Upon integrating this composite as a gain medium into a DBR microcavity, random laser emission was realized by leveraging the high reflectivity and light field localization capability of the DBR structure (Fig. S2b‒S2e) [64]. Nagao *et al.* used natural and biodegradable materials, citric acid and polylysine, as precursors to synthesize all-organic, pure-blue-light-emitting CDs via a controllable hydrothermal reaction. Coupling these CDs with a planar microcavity composed of two DBRs enabled multi-mode laser emission [65]. This study offers valuable insights for developing environmentally friendly laser gain media free of heavy metals and rare earth elements (Fig. S2f‒S2h). Lu *et al.* successfully prepared highly luminescent red-light-emitting CDs by introducing electron donors and precisely controlling the graphitic nitrogen doping level in non-toxic aliphatic precursors. Surface passivation was subsequently employed to enhance the QY and photostability of the CDs. When these passivated CDs were encapsulated within a DBR device, a single longitudinal mode laser with a *Q*-factor as high as 4600 was achieved [66]. This study advances the development of CD-based solid-state lasers and has spurred further exploration of their potential applications (Fig. S2i‒S2m).

To date, researchers have successfully demonstrated laser emission from CDs in both cavity-free random laser systems and cavity-based configurations, underscoring their potential as alternatives to conventional inorganic semiconductors and organic dye materials. However, the underlying luminescence mechanism of CDs remains insufficiently understood, leaving a lack of definitive theoretical guidance for material design, performance optimization and device integration. This knowledge gap continues to present substantial challenges to the development and application of CD-based lasers.

## GAIN MECHANISM OF CDS

For many years, the lack of advanced purification techniques combined with the inherent structural complexity of CDs has led to persistent uncertainty regarding the precise origin of their luminescence. Consequently, establishing a definitive relationship between structure and luminescent properties has remained difficult. This ambiguity has notably constrained the rational design and performance optimization of CD-based laser gain materials [67,68]. Within this context, elucidating the intrinsic gain mechanism of CDs, developing accurate laser emission models, refining their band structure characteristics, and identifying key factors influencing laser emission have become central challenges in advancing high-performance CD laser systems. To address these issues, this section systematically explores the laser generation mechanism of CDs by examining their structural features and band structures while also drawing comparisons with other solution-processable gain materials. This integrated, multi-faceted analysis is expected to reveal the fundamental principles underlying laser gain in CDs.

**Figure 4.**
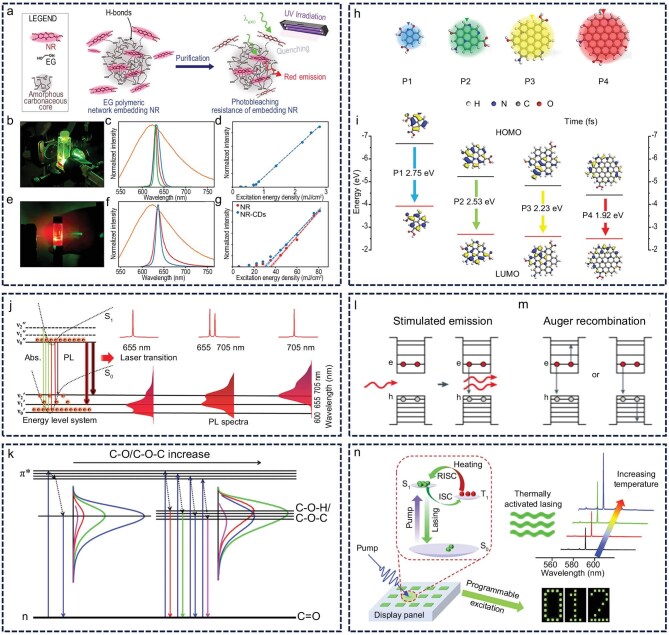
Gain mechanism. (a‒g) Laser emission from dye-derived CDs via luminescent molecular states. NR, neutral red; EG, ethylene glycol. Reprinted with permission from ref [70]. Copyright 2023 American Chemical Society. (h and i) The size effect of CDs leads to a redshift in fluorescence emission. Reprinted with permission from ref [74]. Copyright 2022 Wiley. (j) Six-level energy-level model from dual-wavelength tuning CDs. Reprinted with permission from ref [79]. Copyright 2024 Elsevier Inc. (k) Energy-level diagrams of λ_ex_-independent/-dependent PL. Reprinted with permission from ref [46]. Copyright 2016 American Chemical Society. (l and m) Stimulated emission and Auger recombination schematic. Reprinted with permission from ref [7]. Copyright 2021 Springer Nature (n) TADF laser emission mechanism. Reprinted with permission from ref [91]. Copyright 2021 American Chemical Society.

To clarify the specific gain mechanism of CDs in laser systems, their multi-layered structures can be first examined. CDs typically comprise an internal, densely packed sp^2^/sp^3^-hybridized carbon core and an outer surface shell enriched with functional groups [69]. The sp^2^/sp^3^ carbon framework within the core serves not only as a scattering center that enhances the interaction between the light field and the gain medium but may also contribute optical gain via its delocalized π-electron system. Meanwhile, the molecular or surface states within the shell act as the primary luminescent centers, sustaining the optical gain required for light amplification through stimulated emission. Madonia *et al.* fabricated CDs with a core–shell architecture using neutral red dye, and achieved red laser emission by leveraging both the scattering effect of the core and the luminescent molecular states in the shell [70]. The laser performance of these CDs surpassed that of the neutral red dye alone, which the authors attributed to a synergistic gain mechanism arising from the carbon core and the dye-functionalized shell layer (Fig. 4a‒g). This core–shell configuration—where the core modulates light field distribution and the shell governs optical gain—confers CDs with nanoparticle-like scattering properties while allowing tunable luminescence through surface chemical modification. Consequently, such structural versatility offers distinct advantages for designing solution-processable microlasers.

The luminescence behavior of CD core–shell structures is also influenced by the degree of carbonization [71]. In highly carbonized CDs, the core develops a dense sp^2^-conjugated carbon domain. The luminescence mechanism in such systems is predominantly governed by the size effect, which involves two interrelated factors: the size of the conjugated domain and the quantum confinement effect. Specifically, as the conjugated domain becomes smaller, the emission wavelength undergoes a blue shift. Furthermore, when the physical dimensions of the material fall below the exciton Bohr radius, the quasi-continuous electronic energy levels become discrete, resulting in a further blue shift in fluorescence emission [72,73]. Gong *et al.* combined density functional theory calculations with experimental observations and inferred that graphite nitrogen domains and sp^2^ carbon domains collaboratively modulate the bandgap of CDs. Their results indicated that as the size of the CDs increases, the bandgap narrows, leading to a red shift in fluorescence emission (Fig. 4h‒i) [74]. Conversely, in less carbonized CDs, the carbon skeleton in the core remains underdeveloped, and the shell layer—enriched with specific molecular or surface states—plays the dominant role. In such cases, luminescence primarily arises from electronic transitions associated with these molecular or surface states [75]. Li’s team synthesized blue-light-emitting CDs dominated by surface amide groups via a simple condensation reaction between carboxyl and amine precursors. These amide groups were found to be critical in preventing fluorescence quenching in the solid state [76]. This finding suggests that regulating the distribution of surface states in the shell layer of low-carbonized CDs can enable the construction of CD materials with tailored luminescence properties. Additionally, in less carbonized CDs, the cross-linking enhanced emission (CEE) effect suppresses the rotation and vibration of luminescent centers, thereby promoting cooperative enhancement of PL [77]. Yang *et al.* investigated the impact of the CEE effect on luminescence by constructing an appropriate CD model system [78]. Their experiments revealed that introducing methyl groups into the precursors increases the chain spacing within the CDs, which in turn weakens the CEE effect. This study elucidates the concept of CEE and offers a generalizable strategy for tuning CD luminescence. These luminescence mechanisms provide a theoretical foundation for optimizing the laser performance of CDs. Clarifying the dominant luminescence mechanism and identifying the nature of the luminescent centers are essential for the targeted design of gain-enhancing strategies.

To achieve stable laser emission, the gain medium must contain luminescent centers capable of establishing population inversion. The four-level system, a representative framework for realizing population inversion, has been widely employed in the development of high-performance lasers. However, in complex applications such as optical communication and multi-parameter optical sensing, single-wavelength emission cannot meet the system’s requirement for wavelength multiplexing. Moreover, the conventional four-level model often fails to accurately describe the intricate electronic transition processes involved. To overcome these limitations, the six-level system extends the four-level framework by introducing two additional relaxation channels, forming a more elaborate energy transfer pathway that supports simultaneous dual-wavelength laser output. Lu *et al.* demonstrated tunable dual-wavelength laser emission within a single laser system by constructing a six-level energy-level model [79]. This structure enables population inversion at two distinct wavelengths, thereby facilitating dual-wavelength lasing output (Fig. 4j). The dual-wavelength lasing mechanism established by the six-level system not only enables programmable all-optical logic gate operations but also demonstrates the feasibility of integrating novel CD lasers into optical computing chips. During the construction of the energy level system, competitive transitions among non-target energy levels can hinder population inversion at specific levels, thereby increasing the laser emission threshold. Liu’s research group found that the absence of amplified spontaneous emission (ASE) in certain CDs was caused by additional energy levels introduced by specific structural groups, which disrupted effective population inversion (Fig. 4k) [46]. This study illustrates that precisely regulating the luminescent properties of CDs can minimize particle dissipation through non-lasing energy levels, thus enhancing population inversion. This finding offers a clear pathway for lowering the lasing threshold and underscores the importance of targeted energy level control during material design to improve laser performance.

Although the laser gain mechanism of CDs is not yet fully understood, their material properties incorporate features of both inorganic quantum dots and organic fluorescent molecules. Consequently, strategies used to enhance the laser performance of these two material classes can be adapted for CDs [80,81]. By examining the carrier dynamics of inorganic quantum dots during stimulated emission and the regulation of triplet excitons in organic molecular systems, a theoretical framework and practical insights can be developed to optimize the laser performance of CDs.

From the standpoint of inorganic quantum dots, Auger recombination represents the key bottleneck limiting improvements in laser efficiency [82,83]. This non-radiative transition process markedly suppresses stimulated emission and hinders the radiative recombination of carriers (Fig. 4l and m) [7]. Guo *et al.* incorporated phosphorus (P) atoms into nitrogen-doped CDs. This modification dramatically altered the types and densities of defects, as well as the surface trap states, thus regulating the carrier dynamics and enhancing the optical and photoelectric properties of the CDs [84]. This result demonstrates the potential to improve the laser performance of CDs through the study of carrier dynamics. Beard *et al.* investigated carrier recombination processes in CH_3_NH_3_PbBr_3_ and CH_3_NH_3_PbI_3_, demonstrating that the higher exciton binding energy of lead-bromide-based perovskites results in an Auger recombination rate constant one order of magnitude greater than that of their iodide-based counterparts [85]. Xiong *et al.* notably suppressed the Auger recombination process by coating a CdS shell onto the core of CsPbBr_3_ quantum dots [86]. When these modified quantum dots were integrated into a microtube resonator, they enabled low-threshold laser emission. These findings suggest that, for CD materials, mitigating Auger recombination—either by modulating exciton binding energy or applying surface passivation—may offer a viable route to improving laser performance.

Studies on exciton dynamics in organic molecular systems have shown that the accumulation of triplet excitons considerably impairs laser performance, primarily because triplet–triplet absorption introduces additional optical losses [87,88]. Based on this understanding, the central challenge in improving CD laser performance lies in effectively limiting the buildup of triplet excitons. Two complementary strategies can be employed to address this issue: (i) suppressing the intersystem crossing (ISC) between singlet and triplet states through molecular structure design; and (ii) utilizing the thermally activated delayed fluorescence (TADF) mechanism. By avoiding the use of heavy atoms or transition metals—thereby minimizing spin–orbit coupling—the ISC process can be effectively suppressed, which in turn stabilizes singlet excitons [89,90]. Importantly, the TADF process facilitates the conversion of triplet excitons back into singlet states via a reverse ISC (RISC) process induced by thermal activation, allowing for efficient recycling of triplet excitons. Zhou *et al.* reported the fabrication of self-assembled microspheres uniformly doped with TADF molecules. These microspheres employed the RISC process to convert triplet excitons into singlet excitons, thereby directly facilitating population inversion in the gain medium and enabling thermally activated lasing [91]. This study offers valuable guidance for the development of miniaturized lasers with tailored functionalities (Fig. 4n). Hu *et al.* immobilized CDs within a rigid crystalline network through ionic bonding, imparting TADF characteristics to the material. Upon heating, the structure converts triplet excitons into singlet states via thermal activation, resulting in fluorescence emission and exhibiting thermally enhanced luminescence behavior [92]. This strategy not only suppresses non-radiative exciton losses but also improves luminescence efficiency, providing a structural regulation approach for the design of high-gain, low-threshold CD laser materials.

## STRATEGIES FOR IMPROVING CD LASER PERFORMANCE

Solution-processable CDs have shown progress in advancing the performance of miniaturized lasers. However, their laser output remains limited compared to conventional systems such as organic dyes and inorganic quantum dots. This gap largely stems from the lack of a comprehensive theoretical foundation and clearly defined technical strategies to guide further performance improvements. To address this fundamental scientific issue, this chapter explores strategies for optimizing CD laser performance from three main perspectives: tuning the fluorescence characteristics of CDs; applying structural functionalization and surface modifications; and designing novel resonator architectures. This systematic, multi-level investigation is expected to establish solid theoretical guidance and practical methodologies for improving the laser output performance of CDs.

### Regulation of fluorescence behavior

As a laser gain medium, the fluorescence characteristics of the material play a crucial role in determining its laser emission performance. Key parameters influencing this behavior include the fluorescence QY (φ), fluorescence lifetime (τ), full width at half-maximum (FWHM) of the emission spectrum, and photothermal stability [27,93,94]. Table S2 summarizes representative fluorescence parameters of CD materials employed as solution-processable gain media in this field.

**Figure 5.**
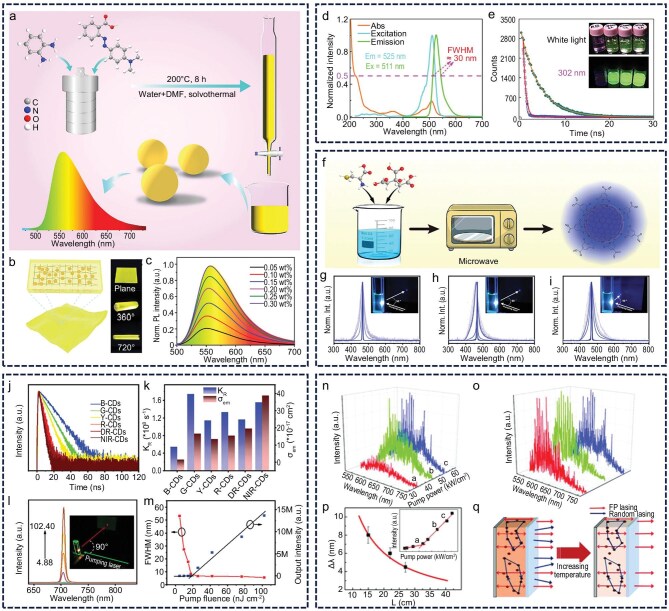
Regulation of fluorescence characteristics. (a‒c) High-efficiency yellow CDs retaining 85% PLQY in PS solid film. DMF, dimethylformamide. Reprinted with permission from ref [100]. Copyright 2022 Wiley. (d and e) Efficient silicon-based CDs with near-unity QY and narrow FWHM. Reprinted with permission from ref [104]. Copyright 2018 American Chemical Society. (f‒i) Optimized precursor ratio toward low-threshold lasing in blue CDs with high PLQY. Reprinted with permission from ref [95]. Copyright 2023 Wiley. (j‒m) Stimulated emission cross-section influences the laser threshold of different CDs. B-CD, blue-emitting CD; G-CD, green-emitting CD; Y-CD, yellow-emitting CD; R-CD, red-emitting CD; DR-CD, deep-red-emitting CD; NIR-CD, near-infrared CD. Reprinted with permission from ref [41]. Copyright 2023 Wiley. (n‒q) High-temperature lasing from CDs leads to changes in the laser characteristics. Reprinted with permission from ref [96]. Copyright 2021 Royal Society of Chemistry.

A higher fluorescence QY directly facilitates the radiative release of energy from excited-state excitons. This not only increases the density of excitons in the excited state to promote population inversion but also substantially reduces thermal buildup by suppressing non-radiative transition pathways, thereby maintaining the thermal stability of the gain medium under continuous excitation. In parallel, a shorter fluorescence lifetime indicates that excited-state electrons can rapidly return to the ground state, which supports coherent photon accumulation in fast optical resonant circuits and enables efficient optical amplification [99]. Shan *et al.* successfully synthesized yellow CDs with a fluorescence QY approaching 100%, which retained a luminescence efficiency as high as 85% after being doped into a PS matrix to form a solid-state film (Fig. 5a‒c) [100]. This outcome illustrates that a high QY not only improves the system’s energy conversion efficiency but also markedly suppresses the aggregation-caused quenching effect, thereby offering more favorable conditions for population inversion between excited and ground states. From the perspective of the structure of CDs, large rigid conjugated planar CDs with highly sp^2^-conjugated domains can be prepared through aromatic precursors to achieve high QY fluorescence [55,101]. On the other hand, increasing the carbonization degree of CDs can reduce their fluorescence lifetime, which is conducive to achieving population inversion [102]. A narrow emission spectral FWHM limits extraneous energy-level transition pathways, thereby concentrating the inverted population in a few dominant transitions. This reduces unnecessary competition among resonant modes and minimizes energy loss in non-coherent oscillation modes, ultimately lowering the lasing threshold [103]. Chen *et al.* successfully synthesized organic silicon-based CDs with excellent optical properties using a one-step hydrothermal method. These CDs exhibit a fluorescence QY approaching 100%, a fluorescence lifetime of 4.2 ns, and a narrow FWHM of only 30 nm [104]. Such outstanding optical characteristics enable efficient population inversion under gain conditions, making this material a highly promising laser gain medium (Fig. 5d and e). Since the CDs are composed of sp^2^/sp^3^ hybrid carbon core and a shell containing functional groups, reducing the content of sp^3^ hybrid carbon within the carbon core is conducive to narrowing of the spectrum and achieving a reduction in redundant modes [105].

While parameters such as QY, fluorescence lifetime and emission spectral FWHM are crucial indicators of a material’s general luminescent properties, they cannot directly quantify the ability to achieve ASE. The generation of gain is a fundamental prerequisite for achieving ASE. The gain coefficient (*g*), which quantifies the light amplification capability during propagation through the laser medium, is directly proportional to the product of the stimulated emission cross-section (σ_em_) and the population inversion density [106]. Accordingly, the stimulated emission cross-section is a critical parameter in determining laser output performance and is mathematically expressed as


\begin{eqnarray*}
{{\mathrm{\sigma }}}_{{\mathrm{em}}}(\lambda) = \frac{{ {\lambda }^4{E}_f\left( \lambda \right)}}{{8{\mathrm{\pi }}{n}^2\left( \lambda \right){\mathrm{c}}{\tau }_f}},
\end{eqnarray*}

where λ represents the emission wavelength, ${E}_f( \lambda )$ denotes the fluorescence quantum distribution, *n* is the refractive index of the material, c denotes the speed of light, and ${\tau }_f$ represents the radiative lifetime. The fluorescence QY is defined as $\varphi = \mathop \smallint \nolimits_0^\infty {E}_f( \lambda )d\lambda $ [107,108]. Therefore, a high fluorescence QY and a short fluorescence lifetime enhance the stimulated emission cross-section, thereby improving the gain characteristics of the material. Lu *et al.* synthesized a series of low-toxicity blue CDs by modulating the proportion of aliphatic precursors. When the feed ratio of citric acid to cysteine was 2:1, the resulting CDs exhibited notably higher fluorescence QY and calculated stimulated emission cross-section compared to CDs produced with other feed ratios [95]. Laser performance tests further demonstrated that the laser threshold of this material was substantially lower than that of CDs prepared using other feed ratios (Fig. 5f‒i). In May of the same year, the team successfully fabricated CDs with emission spanning from blue to near-infrared. Further investigations revealed that the stimulated emission cross-section of blue-light-emitting CDs was over an order of magnitude lower than that of CDs emitting at other wavelengths, resulting in a laser threshold more than 10 times higher than that of materials within the same system (Fig. 5j‒m) [41]. This elevated threshold was primarily attributed to the relatively low QY and longer fluorescence lifetime of the blue CDs. These findings further confirm that regulating fluorescence properties to optimize the stimulated emission cross-section is an effective strategy for enhancing laser performance.

The photothermal stability of fluorescence in gain media plays a vital role in determining their amplification performance. High photothermal stability enables reliable and sustained emission under intense optical pumping by minimizing pump-induced degradation of the material. Notably, the red-emitting CD material reported by Zhang *et al.* maintains stable laser output even at 250°C, during which a transition from random lasing to F–P lasing is observed. This shift is attributed to a decline in gain at elevated temperatures, rendering the medium incapable of supporting random lasing (Fig. 5n‒q) [96]. As such, temperature sensitivity may restrict the operational efficacy of gain materials, whereas outstanding photothermal stability strengthens their resistance to thermal quenching and inhibits heat buildup from non-radiative decay processes. Composed of sp^2^-conjugated domains modified by sp^3^-hybridized carbon groups, CDs are fundamentally nanoparticles whose stability is principally governed by the sp^2^ carbon skeleton. Therefore, the stability of CDs can be effectively enhanced by increasing the degree of carbonization [109].

### Structural functionalization

Structural functionalization is an effective strategy for minimizing optical losses in CDs during the gain process. This reduction primarily results from the optimized design of core–shell architectures, which is achieved through precise control of the chemical microenvironment during both synthesis and post-synthetic processing. Current approaches to this regulation focus on three key aspects.

**Figure 6.**
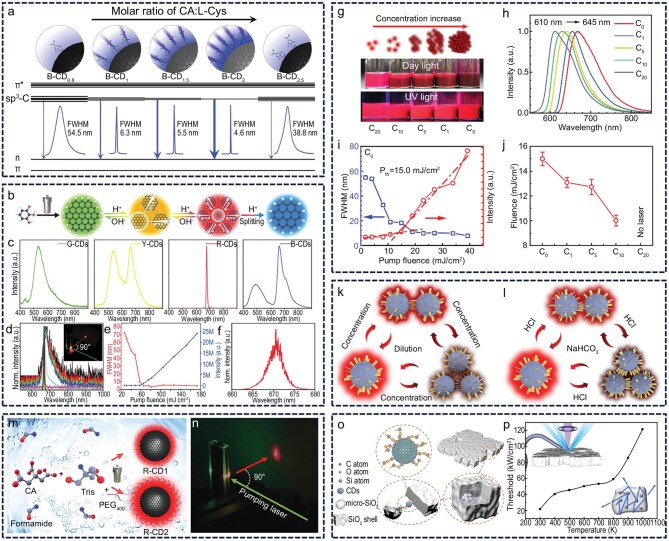
Structural functionalization. (a) Blue-emitting CD lasing mechanism. CA, citric acid; L-Cys, l-cysteine. Reprinted with permission from ref [95]. Copyright 2023 Wiley. (b‒f) Controlling aggregation-induced red laser emission in CDs via pH modulation. G-CD, green-emitting CD; Y-CD, yellow-emitting CD; R-CD, red-emitting CD; B-CD, blue-emitting CD. Reprinted with permission from ref [48]. Copyright 2023 Wiley. (g‒j) Laser threshold control by modulating the aggregation state via CD concentration. Reprinted with permission from ref [97]. Copyright 2024 Elsevier Ltd. (k and l) Dual-wavelength tunable laser emission from CDs mediated by concentration and pH. Reprinted with permission from ref [79]. Copyright 2024 Elsevier Inc. (m and n) Surface functionalization of CDs with chemical-passivation-based coatings for ASE threshold reduction. PEG, polyethylene glycol. Reprinted with permission from ref [66]. Copyright 2021 Wiley. (o and p) Enhance the laser performance of CDs through physical isolation of the SiO_2_ shell layer. Reprinted with permission from ref [113]. Copyright 2024 Elsevier B.V.

The density of sp^3^-hybridized carbon-related excited-state energy levels has been shown to correlate positively with the presence of C–N, C–O and C–S functional groups [110]. A high density of these sp^3^-hybridized levels facilitates the uniform aggregation of electrons, thereby increasing the population difference between the excited and ground states in a four-level system and promoting population inversion. Leveraging this mechanism, the optical losses associated with sp^3^-hybridized carbon-related excited-state levels can be mitigated by finely tuning the relative proportions of heteroatom-containing groups in the CD precursors. For example, Lu and co-workers demonstrated that increasing the ratio of aliphatic precursors results in a more concentrated spatial distribution of sp^3^-associated excited-state energy levels [95]. Consequently, the radiative transition rate of CDs is notably enhanced. This synergistic effect enables more efficient conversion of excited-state energy into radiative emission, ultimately reducing the laser threshold by half (Fig. 6a).

By adjusting the concentration and pH of the synthesis system, the formation of surface states on CDs can be effectively controlled, thereby influencing their aggregation behavior and modulating their gain characteristics [111,112]. Lu *et al.* regulated the protonation of CD surfaces by tuning the pH of the reaction environment, which in turn affected their aggregation state. This aggregation expanded the π-conjugated domain, resulting in altered fluorescence properties [48]. The aggregated structure facilitated laser emission in the red-light region, attributed to enhanced local gain. However, excessive acid addition disrupted intermolecular interactions, destabilizing the local structure and reducing gain to levels insufficient for sustaining laser emission (Fig. 6b‒f). In a separate study, the same team examined how aggregation influences laser performance by varying CD concentration. They found that increasing concentration intensified aggregation, which promoted exciton–exciton annihilation, markedly suppressed radiative recombination efficiency, and ultimately elevated the laser threshold [97]. Conversely, at low concentrations, the gain medium lacked sufficient density to establish effective light-scattering and amplification pathways, making sustained laser resonance difficult (Fig. 6g‒j). Building on these findings, the team successfully realized dual-wavelength tunable laser emission by simultaneously regulating both the concentration and pH of the CD solution, exploiting their distinct roles in inducing aggregation. Under acidic conditions, protonation promoted the formation of an intermolecular hydrogen-bonding network among 5,14-dihydroquinoxalino[2,3-b]phenazine fluorophores on the CD surface, which induced coplanar aggregation and mitigated the aggregation-induced quenching effect (Fig. 6k and l) [79].

Moreover, targeted surface coating treatments can effectively mitigate surface defects and reduce light losses arising from exciton transitions to defect energy levels. These coatings also suppress exciton absorption in the triplet state. Through a combination of physical isolation and chemical passivation, the stability of luminescent centers is considerably improved. Lu *et al.* applied chemical-passivation-based surface coatings to functionalize CDs, successfully lowering their ASE threshold and enabling laser emission within a DBR cavity. This approach not only reduced the influence of surface defect states but also substantially enhanced the fluorescence QY and photothermal stability of the CDs, thereby improving their overall laser performance (Fig. 6m and n) [66]. In a separate study, Zhang *et al.* physically isolated CDs by constructing an SiO_2_ shell layer. This architecture not only enhanced the optical gain characteristics but also provided thermal insulation, effectively suppressing thermal quenching and allowing laser output to be sustained at temperatures as high as 1000 K (Fig. 6o and p) [113]. Collectively, these findings underscore that surface coating strategies can improve the photothermal stability of CDs, minimize light loss during laser emission and enhance laser performance.

### Design of novel resonant cavity

In addition to optimizing the gain medium, the design of resonators is a critical factor in improving laser performance. The gain medium determines the inherent capacity for laser generation, whereas the resonator translates this potential into high-efficiency laser output by modulating photon feedback and minimizing optical losses. Advanced resonator configurations not only suppress light scattering but also enhance beam quality [114,115]. Therefore, continued progress in resonator design and fabrication is essential to support the miniaturization of laser systems. At present, resonator designs for CD-based lasers remain largely constrained to conventional architectures such as F–P cavities, WGM resonators and DBR cavities. While these structures are well established, they typically exhibit relatively high optical losses and limited control over mode properties, which consequently restrict improvements in laser output efficiency. For instance, F–P cavities often suffer from significant diffraction losses and require high precision in mirror alignment. WGM resonators, though capable of high *Q*-factors, are sensitive to environmental disturbances and surface imperfections.

**Figure 7.**
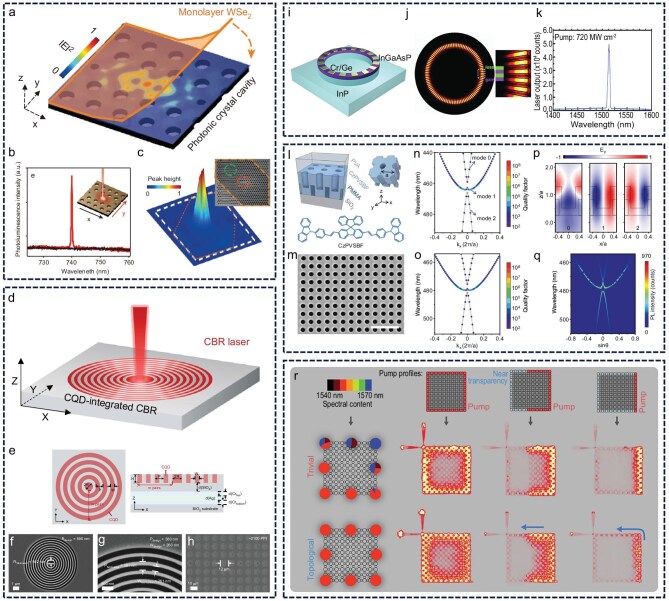
Design of a novel resonant cavity. (a‒c) Photonic crystal cavity. Reprinted with permission from ref [116]. Copyright 2015 Spring Nature Limited. (d‒h) CBR. CQD, colloidal quantum dot. Reprinted with permission from ref [117]. Copyright 2025 Spring Nature. (i‒k) PT-symmetric laser system. Reprinted with permission from ref [114]. Copyright 2014 AAAS. (l‒q) BIC cavity. Reprinted with permission from ref [118]. Copyright 2025 Spring Nature. (r) Topological laser arrays. Reprinted with permission from ref [119]. Copyright 2018 AAAS.

In contrast, emerging micro–nano resonators have demonstrated considerable performance advantages in other material systems. These include photonic crystal cavities, circular Bragg resonators (CBRs), parity–time (PT) symmetry cavity, bound states in the continuum (BIC) and topological microcavities. Such designs enable lower lasing thresholds, greater efficiencies and precise mode control by enhancing light confinement and reducing loss pathways. Xu *et al.* demonstrated an ultralow-threshold nanoscale laser through the incorporation of quantum dots into a photonic crystal cavity. This cavity structure effectively reduced the lasing threshold of the emitter, enabling a compact laser system that operates with minimal power consumption and supports ultrafast modulation. (Fig. 7a–c) [116]. In a separate study, Wang *et al.* integrated high-quality colloidal quantum dots into a CBR and successfully achieved low-threshold laser emission. Compared with traditional DBR vertical-cavity surface-emitting lasers, this device exhibited a substantially reduced lasing threshold and demonstrated stable operation for up to 1000 h (Fig. 7d–h) [117]. Zhang *et al.* designed a PT-symmetric laser system based on micro-ring cavities, which can freely control the resonant modes. This system can achieve stable single-mode laser emission by strictly controlling the interaction between gain and loss (Fig. 7i‒k) [114]. Zhao *et al.* designed a 2D organic semiconductor metasurface to enable room-temperature, exciton–BIC strong-coupling-controlled, topologically reconfigurable polariton condensates. The system’s ultralow lasing threshold arises from the synergistic interaction between Frenkel excitons and infinite-Q bound states in the BICs (Fig. 7l‒q) [118]. Mercedeh *et al.* developed a 2D microlaser array based on all-dielectric topological insulators, enabling high-performance single-mode lasing through topologically protected edge modes that are immune to scattering. The slope efficiency of this system exceeds that of topologically trivial counterparts, reflecting its superior lasing performance. Additionally, it is fully compatible with existing semiconductor manufacturing processes and offers a promising route for the development of next-generation topological laser arrays with high coherence and efficiency (Fig. 7r) [119]. Integrating these advanced resonator structures with CD gain media will provide essential technical support for the continued development of compact CD-based lasers.

## APPLICATION PROGRESS AND CHALLENGES

In recent years, the growing demand for miniaturization, flexibility and integration has propelled research on solution-processable miniaturized lasers to the forefront of both academic and industrial efforts. However, concerns regarding the toxicity of solution-processable gain media to human health and the environment need to be addressed. This issue has become a major barrier to the practical deployment of such miniaturized laser systems [120,121]. Against this backdrop, CDs—owing to their unique optical properties, excellent stability and inherent biocompatibility—have emerged as promising candidates for next-generation laser gain media. Moreover, the cost-effectiveness and simple processability of CDs further support their potential for broad market application in miniaturized laser technologies. Even though the application of CD-based lasers remains in an early exploratory stage, considerable challenges persist in areas such as device performance optimization and process integration [39]. Nevertheless, continued advancements in CD material chemistry, along with progress in micro–nano fabrication techniques, are expected to unlock the potential of CD-based miniaturized lasers in terms of cost-effectiveness, optical properties and biology, and to continuously integrate them into high-end applications such as flexible electronics, implantable medical devices and environmentally sustainable photovoltaic systems.

**Figure 8.**
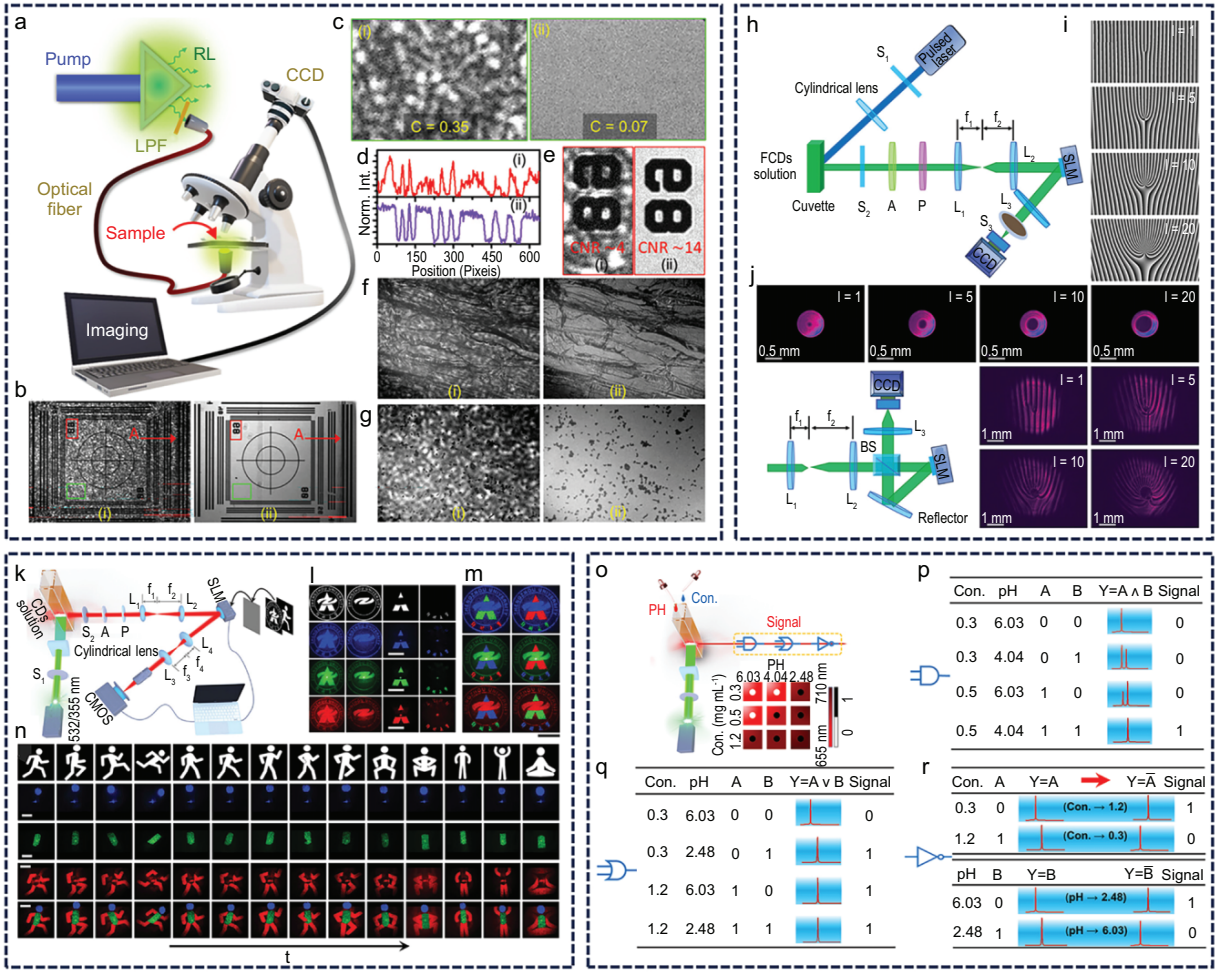
CD laser applications: (a‒g) Microscopic imaging system with CD-based random laser sources. CCD, charge coupled device; LPF, low-pass filter. Reprinted with permission from ref [50]. Copyright 2024 American Chemical Society. (h‒j) CD-based high-purity vortex beam laser. FCD, full-color CD; SLM, spatial light modulator; BS, beam splitter. Reprinted with permission from ref [98]. Copyright 2025 Wiley. (k‒n) Speckle-free color laser imaging using full-color CD lasers. CMOS, complementary metal-oxide-semiconductor. Reprinted with permission from ref [41]. Copyright 2023 Wiley. (o–r) Laser logic gates: (o) schematic, (p) AND, (q) OR, (r) NOT truth tables. Reprinted with permission from ref [79]. Copyright 2024 Elsevier Inc.

Owing to their exceptional wavelength tunability, CD-based miniaturized lasers have been primarily explored for applications in optical imaging and basic logic gate design. In comparison with traditional lasers, random lasers reduce the spatial coherence of emitted light through multiple scattering mechanisms, effectively suppressing speckle artifacts and enhancing imaging quality. This feature offers notable advantages for biomedical imaging [122,123]. Messina *et al.* successfully integrated CD-based random laser sources into a microscopic imaging system, overcoming the speckle effects typically associated with the high spatiotemporal coherence of conventional laser illumination [50]. This integration enabled clear visualization of high-resolution features, such as cell boundaries and subcellular structures, resulting in a marked improvement in imaging quality (Fig. 8a‒g). CD lasers also exhibit excellent biocompatibility and stability, with their low toxicity supporting the feasibility of long-term *in vivo* observation. This capability positions CD lasers as a promising light source for cross-scale imaging applications, including *in situ* detection of pathological tissues and defect analysis in micro–nano devices. Recognizing the advantages of vortex beams in high-capacity optical communication, optical micromanipulation and quantum information processing, Lu *et al.* converted rhodamine B precursors into low-biotoxicity CDs and constructed efficient laser devices. Coupled with a spatial light modulator, CD lasers were used to generate high-purity vortex beams featuring central phase singularities and well-defined ring-shaped intensity distributions [98]. Compared with traditional solution-processable lasers, this system demonstrated superior topological charge controllability and beam shape stability, thereby confirming the feasibility of generating structured lasers at the micro–nano scale within the CD platform (Fig. 8h‒j). This advancement not only expands the light-field control capabilities of carbon-based materials but also introduces a new technological route for the design of micro–nano photonic devices and optical information encoding. Furthermore, Lu *et al.* achieved speckle-free color laser imaging using full-color CD lasers and demonstrated dynamic video displays through programmed sequential control, validating their potential for use in programmable optical displays (Fig. 8k‒n) [41]. Their study advanced not only the imaging applications of CD lasers but also the integration of these systems into optoelectronic devices. By constructing optical logic gates based on dual-wavelength tunable CD lasers, the team provided preliminary validation of the application potential of CD materials in all-optical programmable circuits. This progress offers a valuable technical reference for the future development of CD-based optical computing and photonic integrated chips (Fig. 8o‒r) [79].

**Figure 9.**
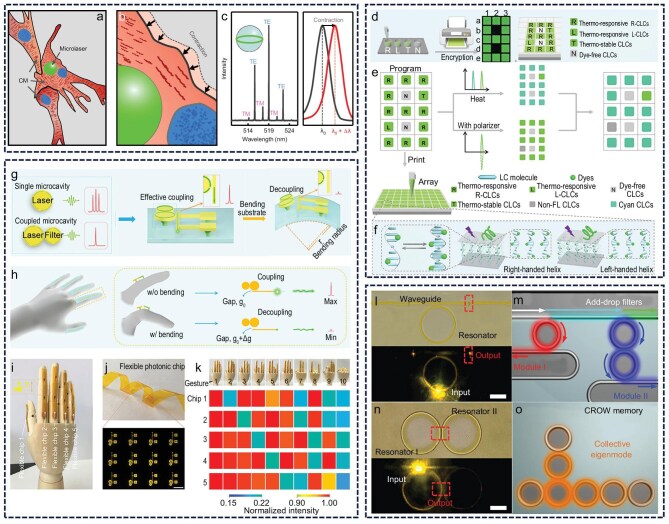
Potential applications. (a‒c) Cell laser: (a) 3D schematic with myofibrillar tissue, (b) contractile movement (CM) visualization, (c) multi-modal spectra with magnification. TM, transverse magnetic; TE, transverse electric. Reprinted with permission from ref [127]. Copyright 2020 Springer Nature Limited. (d‒f) Information encryption: (d) microtemplate printing with encoding ink, (e) wavelength-tunable CP lasing encryption and decryption, (f) dye-doped cholesteric liquid crystal (CLC) helix superstructures. Reprinted with permission from ref [128]. Copyright 2022 Wiley. (g‒k) Mechanical sensing: (g) coupled microdisk-cantilever sensor design, (h) gesture recognition principle, (i) model hand, (j) flexible photonic chip, (k) gesture signal color map. Reprinted with permission from ref [129]. Copyright 2021 AAAS. (l‒o) Integrated circuit: (l) microscopy image of a printed microring resonator, (m) schematic of an as-printed add-drop filter, (n) microscopy image of coupled resonators, (o) schematic of printed coupled resonator optical waveguide. Reprinted with permission from ref [130]. Copyright 2015 AAAS.

Leveraging the green and non-toxic nature of CD materials, along with their advantages in solution processing, these materials hold considerable promise for miniaturized lasers despite ongoing needs for performance optimization. Their distinctive optical properties make them highly suitable candidates for miniaturized laser applications, especially in fields with strict safety and micro–nano compatibility requirements [124–126]. Gather *et al.* successfully integrated microcavity whispering-gallery-mode lasers into cardiac cells, enabling real-time 3D monitoring of cardiac contraction dynamics at single-cell resolution. The high sensitivity of these lasers allows long-term tracking of drug responses, *in vivo* organ contractility and tissue-scale mechanical changes, while also revealing the impact of myofibrillar protein density on contractility (Fig. 9a‒c) [127]. This technology establishes a foundation for non-invasive biosensing based on micro–nano lasers, facilitating precise monitoring of multiple physiological parameters at the cellular level. Zhao *et al.* proposed a multi-dimensional information encryption strategy using circularly polarized (CP) microlaser arrays, markedly enhancing anti-counterfeiting security by exploiting the wavelength tunability and hidden polarization states of thermally tuned liquid crystals (Fig. 9d‒f) [128]. Miniaturized cavity lasers, distinguished by their extremely narrow emission peaks and excellent excitation responses, exhibit superior performance as sensing signal sources through high recognition accuracy. Zhao *et al.* developed a flexible artificial skin system based on organic single-mode laser arrays, wherein local miniaturized lasers achieve precise recognition and mechanically reliable optical signal switching by controlling the coupling and decoupling states of paired microcavities. High-precision gesture recognition is accomplished by analyzing signal outputs from different regions of the laser array (Fig. 9g‒k) [129]. The advantageous properties of miniaturized lasers for sensing also play a vital role in integrated circuits by effectively minimizing energy loss during transmission and storage. Zhao’s team fabricated organic microresonant lasers on flexible chips via solution printing technology, enabling efficient coupling between the optical waveguide and resonator, which yielded low-threshold, high-*Q*-factor devices [130]. This programmable printed photonic circuit successfully integrates filtering and storage functional units, providing new insights into the development of flexible optoelectronic devices (Fig. 9l‒o).

**Figure 10.**
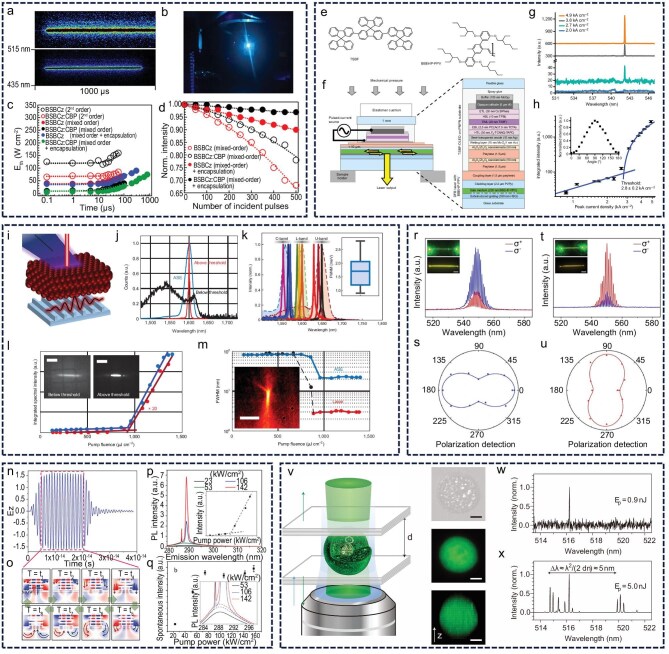
Difficulties and challenges encountered. (a‒d) Continuous laser emission. Reprinted with permission from ref [137]. Copyright 2017 AAAS. (e‒h) Indirect electrical pumping system. Reprinted with permission from ref [138]. Copyright 2023 Spring Nature. (i‒m) Near-infrared II on-chip lasers. Reprinted with permission from ref [143]. Copyright 2021 Spring Nature Limited. (n‒q) Deep ultraviolet plasmonic laser. Reprinted with permission from ref [144]. Copyright 2018 Wiley. (r‒u) Enhancement of the asymmetry factor by laser. Reprinted with permission from ref [147]. Copyright 2024 Wiley. (v‒x) Cell laser using fluorescent proteins. Reprinted with permission from ref [148]. Copyright 2011 Spring Nature Limited.

For CD miniaturized lasers, accelerating practical applications requires two critical technical challenges to be overcome: achieving continuous laser output and transitioning from optical pumping to electrical pumping [131,132]. Compared with pulsed lasers, continuous lasers offer a more compact size and more stable light emission, demanding materials with superior gain properties and enhanced photothermal stability. Achieving continuous laser output is also a key prerequisite for realizing electrical pumping [133,134]. In solution-processable lasers, electrical pumping systems exhibit higher energy utilization efficiency, representing an essential path toward advancing miniaturized laser applications [135,136]. To address these challenges, valuable insights can be drawn from progress in other material systems. Adachi *et al.* demonstrated continuous laser emission using a hybrid resonant cavity that couples organic thin films with DFB structures, highlighting that laser materials with high fluorescence QY, high optical gain and no spectral overlap between the emission peak and the triplet absorption band are favorable for achieving continuous laser output (Fig. 10a‒d) [137]. Direct charge injection into organic semiconductors causes substantial energy loss, undermining the goal of efficient electrical pumping. Samuel *et al.* demonstrated coherent light output below the threshold current by spatially separating the charge injection and gain feedback regions in a coupled system of organic light-emitting diodes and resonant cavities, thereby validating the feasibility of indirect electrical pumping for the first time (Fig. 10e‒h) [138]. This breakthrough introduces a novel 3D device design concept to overcome the electrical pumping bottleneck in CD materials.

A major factor underlying the market potential of CD lasers is their exceptional tunability of fluorescence wavelengths. However, current CD laser emissions are primarily limited to the visible spectrum. Expanding the emission wavelength range is essential to fully realize their application potential. NIR-II (1000–1700 nm) lasers have demonstrated promising applications in biomedical imaging, disease diagnosis and treatment, owing to reduced scattering, deeper tissue penetration and low background fluorescence [139,140]. Deep ultraviolet lasers, notable for their high photon energy, flux and spectral resolution, are critical for laser-driven interference lithography, precision microfabrication, detection and spectroscopy [141,142]. Konstantatos *et al.* achieved NIR-II laser emission in a DFB cavity using solution-processed PbS colloidal quantum dots, enabling tunable emission from 1550 to 1650 nm [143]. Their strategy of threshold reduction through N-doping offers new avenues for developing low-power, highly integrated NIR-II on-chip lasers (Fig. 10i‒m). Tsai *et al.* developed a novel deep ultraviolet plasmonic laser based on a hyperbolic metamaterial cube (super-cavity). This unique subwavelength super-cavity design eliminates the need for long feedback structures typical of traditional plasmonic lasers, enabling deep ultraviolet coherent emission and providing a breakthrough solution for miniaturized deep ultraviolet photonic devices (Fig. 10n‒q) [144].

In addition to addressing material performance challenges in CD-based miniaturized lasers, expanding their functional capabilities remains essential to meet diverse application demands. Current research primarily focuses on optimizing gain performance, whereas relatively limited attention has been given to tuning the properties of the laser output. The development of chiral CD materials for CP laser emission enables control over the laser’s polarization state and angular momentum. This strategy not only increases the information-carrying capacity of the laser but also offers clear advantages compared with traditional CP fluorescence. By leveraging the high coherence of lasers, the asymmetry factor (g-factor) can typically be enhanced by one to two orders of magnitude [145,146]. Zhao *et al.* designed chiral molecules that self-assembled into helical microcrystals, producing strong CP laser emission with an asymmetry factor approaching ∼1.0, representing an amplification of nearly two orders of magnitude (Fig. 10r‒u) [147]. Evidently, CP lasers offer an effective approach to amplifying chiroptical differences. Despite their low toxicity and excellent biocompatibility, CD lasers have seen limited application in biological fields. To fully harness the advantages of CDs, accelerating research into CD lasers for biological applications is a crucial step toward facilitating their practical adoption. Yun *et al.* employed green fluorescent protein as the gain medium and integrated cells into a DBR microcavity to demonstrate the first biocompatible laser (Fig. 10v‒x) [148]. Their pioneering work not only confirmed the feasibility of biological lasers but also provided valuable theoretical foundations for the future development of non-toxic, highly biocompatible CD lasers.

## CONCLUSION AND OUTLOOK

In this review, we discuss how CDs, owing to their simple and cost-effective preparation methods, low toxicity, excellent biocompatibility and superior fluorescence properties, have emerged as promising gain media for solution-processable miniaturized lasers. Notable progress has been made in solution processability and device miniaturization. Recent research primarily focuses on random lasers and classic microcavity lasers such as WGM, F–P and DBR lasers. However, the unclear gain mechanism of CDs remains a major obstacle to further development, compounded by an ambiguous structure–performance relationship that complicates the formulation of effective optimization strategies. The application of CD lasers in complex scenarios remains limited, and current research is far from market-ready applications. Achieving continuous laser output and transitioning from optical pumping to electrical pumping represent key technical challenges in the field. Below, we summarize these challenges and propose potential solutions to advance CD lasers in solution-processable miniaturized laser systems.

The complex structure of CDs results in a luminescence mechanism that remains difficult to unify. As a novel hybrid nanomaterial combining organic and inorganic features, CDs exhibit both the size-dependent effects characteristic of inorganic quantum dots and the molecular-state luminescence typical of organic dyes. This unique dual luminescence mechanism, along with the intricate relationship between laser and fluorescence behaviors, has yet to fully clarify the gain mechanism of CDs. The absence of a clear structure–performance relationship continues to challenge researchers attempting to optimize CDs using traditional methods. Although studies of the gain mechanisms in inorganic quantum dots and organic dyes have provided strategies to enhance specific CD properties, these approaches often focus on optimizing single properties. To improve the overall performance of CDs, deepening the understanding of the intrinsic correlations among these regulatory mechanisms at the structural level is essential. Achieving breakthroughs in practical applications—such as continuous laser output and the transition from optical to electrical pumping—requires detailed molecular-level analysis of the structure–performance relationship. Precise control of the synthesis process, elucidation of the specific structural features of CDs, and systematic investigation of how core-layer size effects and shell-layer molecular states influence gain performance are essential. By developing a thorough understanding of these key factors, a comprehensive structure–performance correlation model can be established, enabling targeted optimization of CD gain properties and providing a robust scientific foundation for the application of CD miniaturized lasers.

Furthermore, applying CD miniaturized lasers in biological systems represents a crucial direction for future development. The core challenge lies in achieving stable optical functionality under biocompatibility constraints—ensuring the physical and chemical stability of CDs as laser gain media while maintaining low toxicity and biodegradability within complex biological microenvironments. From a materials perspective, developing environmentally responsive, biocompatible CDs that preserve core–shell structural integrity during cellular or circulatory processes to guarantee laser performance, while enabling controlled metabolism in response to specific enzymes or pH conditions after clearance from target lesion areas, is crucial. From a device perspective, flexible and integrated miniaturized lasers must be designed to meet operational requirements within living organisms, facilitating *in situ* generation and transmission of optical signals. By addressing these technical challenges, broader biological applications of CDs can be realized, thereby expanding the functional scope of CD lasers.

CD miniaturized lasers have demonstrated initial promise in fundamental applications such as optical imaging and simple optical logic gates. However, substantial challenges remain before these devices can reach market-ready status. In areas including biomedicine, information communication, precision processing and bionic sensing, CD miniaturized lasers hold significant potential. Nevertheless, a pronounced gap exists between current technical capabilities and industrial demands, particularly concerning laser output stability, energy conversion efficiency and operational reliability under real-world conditions. Technically, the realization of continuous laser operation and electrically pumped emission constitute critical bottlenecks. For continuous operation, addressing issues such as heat accumulation and photobleaching during extended use requires both enhancement of intrinsic material stability and optimization of thermal management structures. For electrically pumped lasers, improving carrier mobility and achieving balanced electron–hole recombination are essential priorities.

In summary, driven by the rapid advancement of intelligent micro–nano devices and flexible optoelectronic technologies, the development of CD-based solution-processable miniaturized lasers presents distinct advantages over alternative materials. Continued innovation in the mechanisms and performance of CDs is expected to usher in a new chapter in green nanophotonics.

## Supplementary Material

nwaf426_Supplemental_File
