# Supplementary material for: Current status and future prospects of non-toxicity carbon-dot-based miniaturized lasers
Source: Natl Sci Rev. 2025 Sep 29;12(11):nwaf426. doi: 10.1093/nsr/nwaf426 (PMC12598612; doi:10.1093/nsr/nwaf426)
Supplement: nwaf426_Supplemental_File [file nwaf426_supplemental_file.docx]

**Table S1.** Key laser performance parameters of solution-processable gain media.

| Gain midia | Wavelength (nm) | Threshold (μJ cm^-2^) | *Q*-factor | *K_R_* (radiation transition rate) (s^-1^) |
| --- | --- | --- | --- | --- |
| Organic dyes | 461-1310 | 0.16 | 10^3^-10^4^ | ~10^9^ |
| Colloidal quantum dots | 450-1950 | 0.39 | 10^3^-10^4^ | ~10^8^ |
| CDs | 315-705 | 1.2 | 10^3^-10^4^ | ~10^8^ |

**
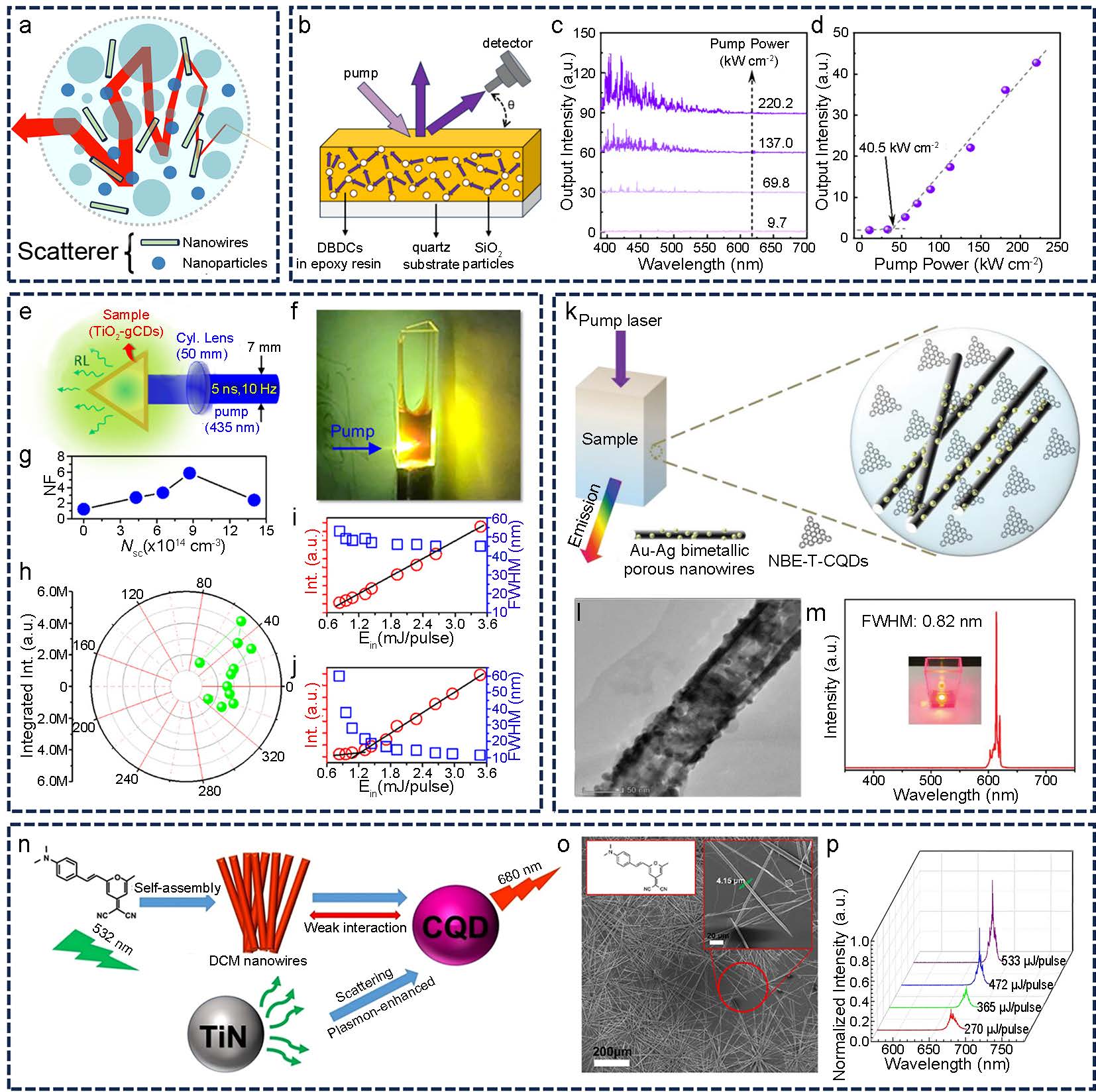
**

**Figure S1.** Random laser with external scatterers. (a) Random laser concept with scatterers. (b–d) Carbon dots-based deep-blue random laser with SiO_2_ particle scatterers. Reprinted with permission from ref [49]. Copyright 2025 American Chemical Society. (e–j) Carbon dots-based random laser with TiO_2_ particle scatterers. Reprinted with permission from ref [50]. Copyright 2024 American Chemical Society. (k–m) Carbon dots-based random laser with Au-Ag nanowire scatterers. Reprinted with permission from ref [51]. Copyright 2018 Wiley. (n–p) TiN nanoparticle-doped Carbon dots-based random laser. Reprinted with permission from ref [52]. Copyright 2022 Optica Publishing Group.


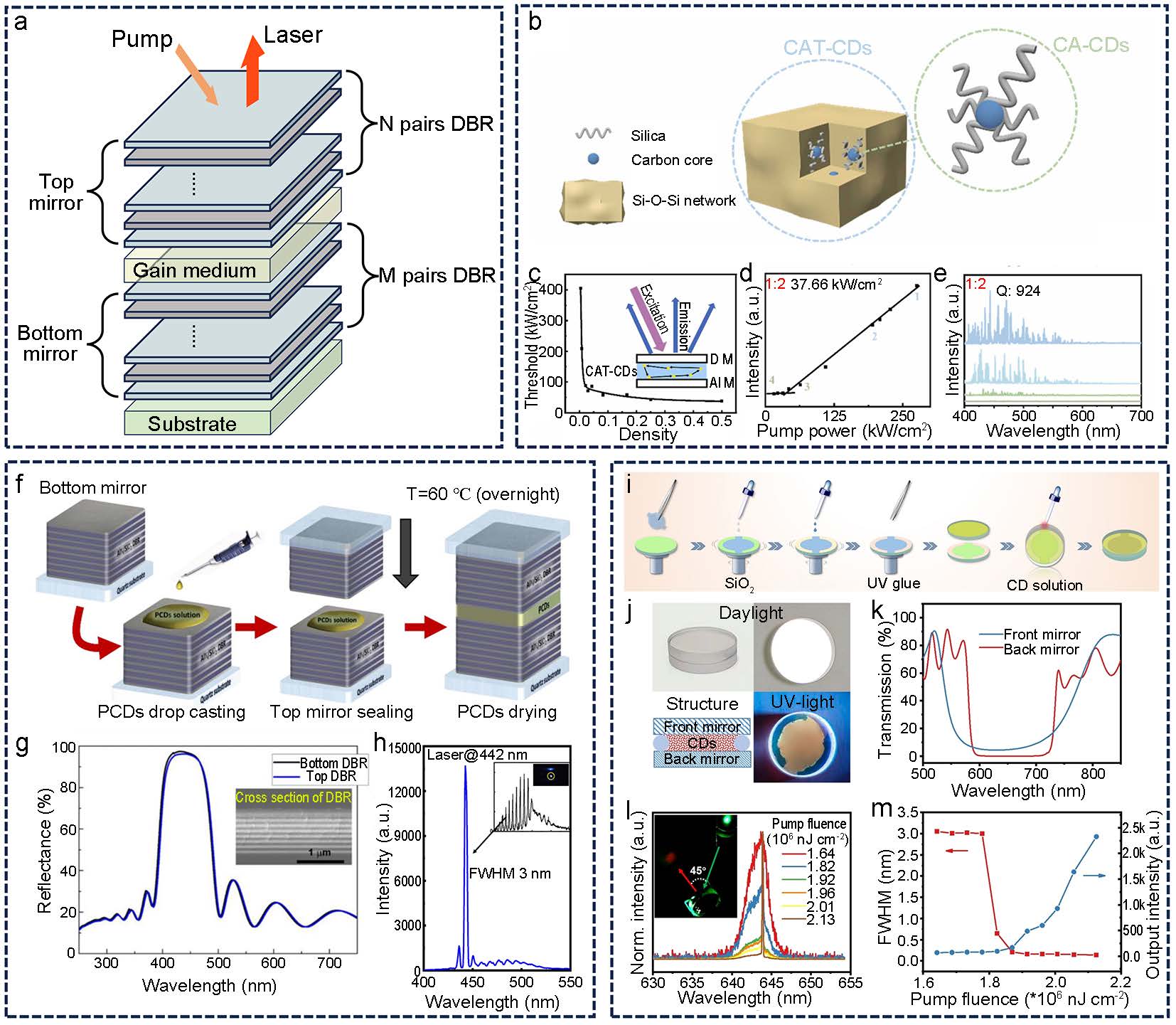


**Figure S2.** DBR laser**.** (a) DBR laser concept. (b) General laser structure. Reprinted with permission from ref [64]. Copyright 2025 Elsevier. (c) Lasing threshold vs. density of CAT-CDs. Reprinted with permission from ref [64]. Copyright 2025 Elsevier. (d) CAT-CD lasing intensity vs. pump power. Reprinted with permission from ref [64]. Copyright 2025 Elsevier. (e) CAT-CD lasing spectra. Reprinted with permission from ref [64]. Copyright 2025 Elsevier. (f) Quartz/DBR/CD sample fabrication. Reprinted with permission from ref [65]. Copyright 2023 American Chemical Society. (g) Reflectance spectra. Reprinted with permission from ref [65]. Copyright 2023 American Chemical Society. (h) Laser emission spectra. Reprinted with permission from ref [65]. Copyright 2023 American Chemical Society. (i) Planar CD microcavity design. Reprinted with permission from ref [66]. Copyright 2021 Wiley. (j) Photographs under daylight and UV light, along with the structural diagram. Reprinted with permission from ref [66]. Copyright 2021 Wiley. (k) DBR mirror transmission spectra. Reprinted with permission from ref [66]. Copyright 2021 Wiley. (l) Mission spectra at different pump fluences. Reprinted with permission from ref [66]. Copyright 2021 Wiley. (m) Intensity and FWHM vs. pump fluence. Reprinted with permission from ref [66]. Copyright 2021 Wiley.

**Table S2.** Fluorescence characteristic parameters of CD materials for solution-processable gain media.

| Wavelength (nm) | QYs (%) | Lifetime (ns) | FWHM (nm) | Reference |
| --- | --- | --- | --- | --- |
| 450 | 38.0 | 4.0 | 75.0 | [46] |
| 526 | 91.2 | 5.2 | 42.4 | [41] |
| 572 | 41.2 | 3.6 | 47.5 | [41] |
| 605 | 51.6 | 3.9 | 70.8 | [41] |
| 714 | 37.9 | 2.4 | 57.6 | [41] |
| 672 | 16.0 | 1.1 | 55.0 | [48] |
| 403 | 61.0 | 5.0 | 35.0 | [49] |
| 535 | 12.0 | 4.5 | 60.0 | [50] |
| 460 | 66.0 | — | 30.0 | [51] |
| 498 | 72.0 | — | 29.0 | [51] |
| 582 | 54.0 | — | 30.0 | [51] |
| 581 | 82.0 | 10.1 | 30.0 | [55] |
| 428 | 20.0 | — | 80.0 | [57] |
| 540 | 36.0 | 10.2 | 115.0 | [60] |
| 490 | 97.2 | 6.9 | 70.0 | [62] |
| 475 | 14.0 | 7.3 | 110.0 | [64] |
| 422 | 49.0 | 1.1 | 60.0 | [65] |
| 655 | 60.6 | 2.0 | 79.0 | [79] |
| 431 | 83.0 | 10.7 | 60.0 | [95] |
| 612 | 66.7 | 7.0 | 90.0 | [96] |
| 630 | 16.0 | 3.1 | 75.0 | [97] |
| 532 | 96.8 | 4.5 | 40.7 | [98] |
| 557 | 58.0 | 4.3 | 41.1 | [98] |
